# From plant survival to thriving: exploring the miracle of brassinosteroids for boosting abiotic stress resilience in horticultural crops

**DOI:** 10.3389/fpls.2023.1218229

**Published:** 2023-07-21

**Authors:** Zhilu Zhang, Zhongyu Chen, Haina Song, Shiping Cheng

**Affiliations:** ^1^ College of Chemistry and Environmental Engineering, Ping Dingshan University, Pingdingshan, Henan, China; ^2^ Henan Province Key Laboratory of Germplasm Innovation and Utilization of Eco-economic Woody Plant, Pingdingshan, Henan, China; ^3^ People’s Park Management Office of Nanyang City Garden and Greening Center, Garden and Greening Center of Nanyang City, Nanyang, Henan, China

**Keywords:** irregular stomatal conductance, oxidative injury, plant defense system, photosynthetic mechanisms, poor growth

## Abstract

Abiotic stresses pose significant threat to horticultural crop production worldwide. These stresses adversely affect plant growth, development, and ultimately declined crop growth, yield and quality. In recent years, plant scientists have been actively investigating innovative strategies to enhance abiotic stress resilience in crops, and one promising avenue of research focuses on the use of brassinosteroids (BRs). BRs are a class of plant hormones that play crucial roles in various physiological processes, including cell elongation, differentiation, and stress responses. They have emerged as potent regulators of plant growth and development, and their role in improving abiotic stress tolerance is gaining considerable attention. BRs have been shown to mitigate the negative effects of abiotic stresses by modulating key physiological and biochemical processes, including stomatal regulation, antioxidant defense, osmotic adjustment, and nutrient uptake. Abiotic stresses disrupt numerous physiological functions and lead to undesirable phenotypic traits in plants. The use of BRs as a tool to improve crop resilience offers significant promise for sustainable agriculture in the face of increasing abiotic stresses caused by climate change. By unraveling the phenomenon of BRs, this review emphasizes the potential of BRs as an innovative approach for boosting abiotic stress tolerance and improving the overall productivity and quality of horticultural crops. Further research and field trials are necessary to fully harness the benefits of BRs and translate these findings into practical applications for crop production systems.

## Introduction

**Figure 1:**
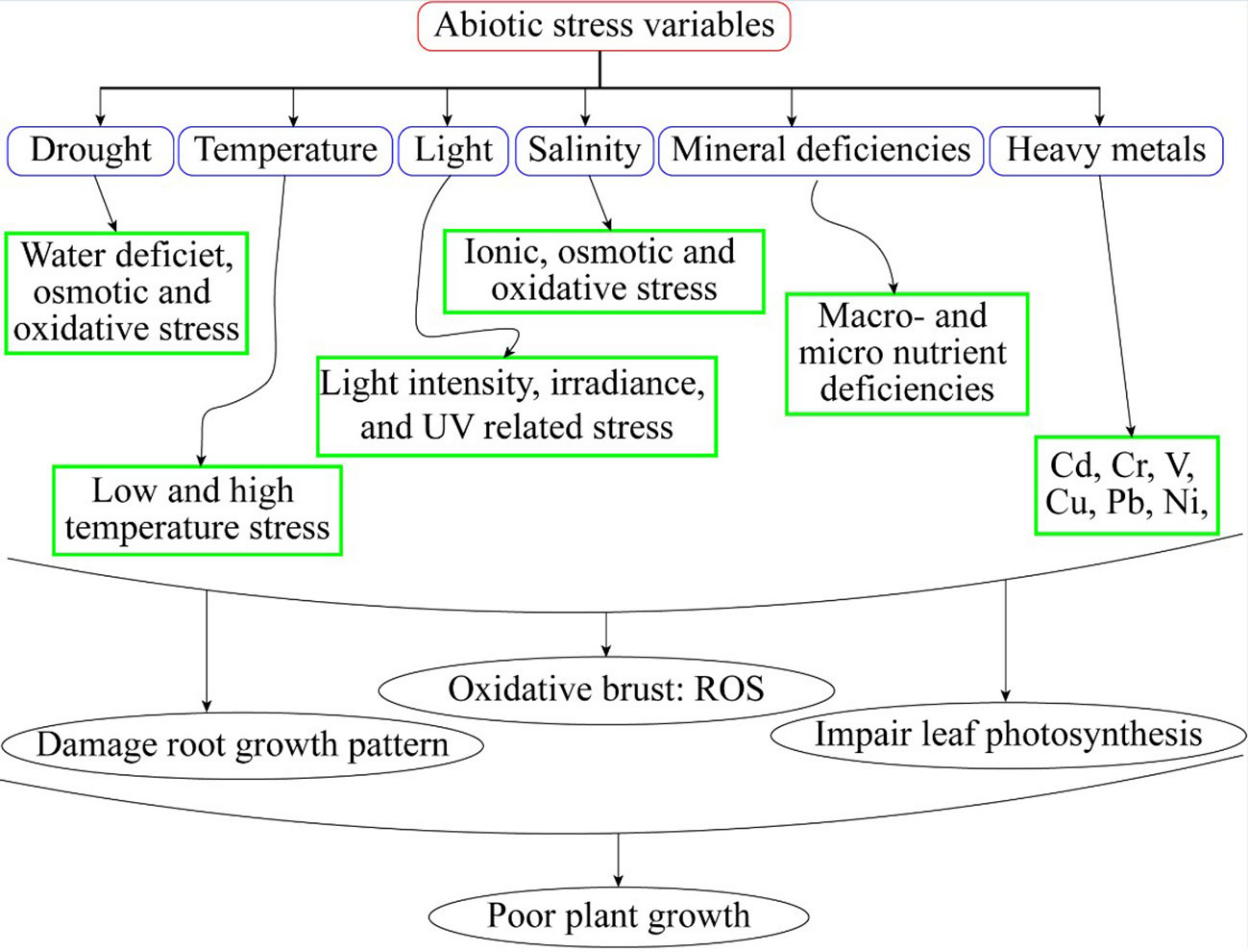
Abiotic stress variables and its effect on plants.

Plants possess intrinsic mechanisms to enhance their tolerance to abiotic stresses (Kanwar et al., 2012). Different physiological and biochemical adjustments are necessary under abiotic stress in horticultural crops (**Figure 1**). These interactions comprise variations in gene regulation, the production of particular proteins and many metabolites, changes in hormonal signaling, and antioxidant capacity (Shahzad et al., 2022). A collection of organic, naturally occurring molecules is known as plant hormones (Rao et al., 2019). Low concentrations of these hormones could influence important plant life cycle activities (Rhaman et al., 2020). Phytohormones are involved in physiological changes and expression of genes under abiotic stresses. Brassinosteroids (BRs), Auxins, gibberellins, cytokinin, abscisic acid, jasmonates, salicylic acid, ascorbic acid, melatonin, and ethylene are only a few examples of the class of naturally occurring compounds known as phytohormones (Sardar et al., 2021). Since the 1940s, they have been utilized in horticultural crops. A few examples of well-studied expressions are ethylene’s encouragement of fruit ripening, auxin and cytokinin control of the cell cycle, gibberellins initiation, seed germination and stem length, and ABA’s maintenance of seed dormancy (Tang et al., 2020). Hormonal processes determine the growth and development of plants (Rabnawaz et al., 2020). Phytohormones involved in signal transduction networks under abiotic stresses resulting in improved growth and yield of horticultural crops (Muhammad et al., 2022).

**Figure 2:**
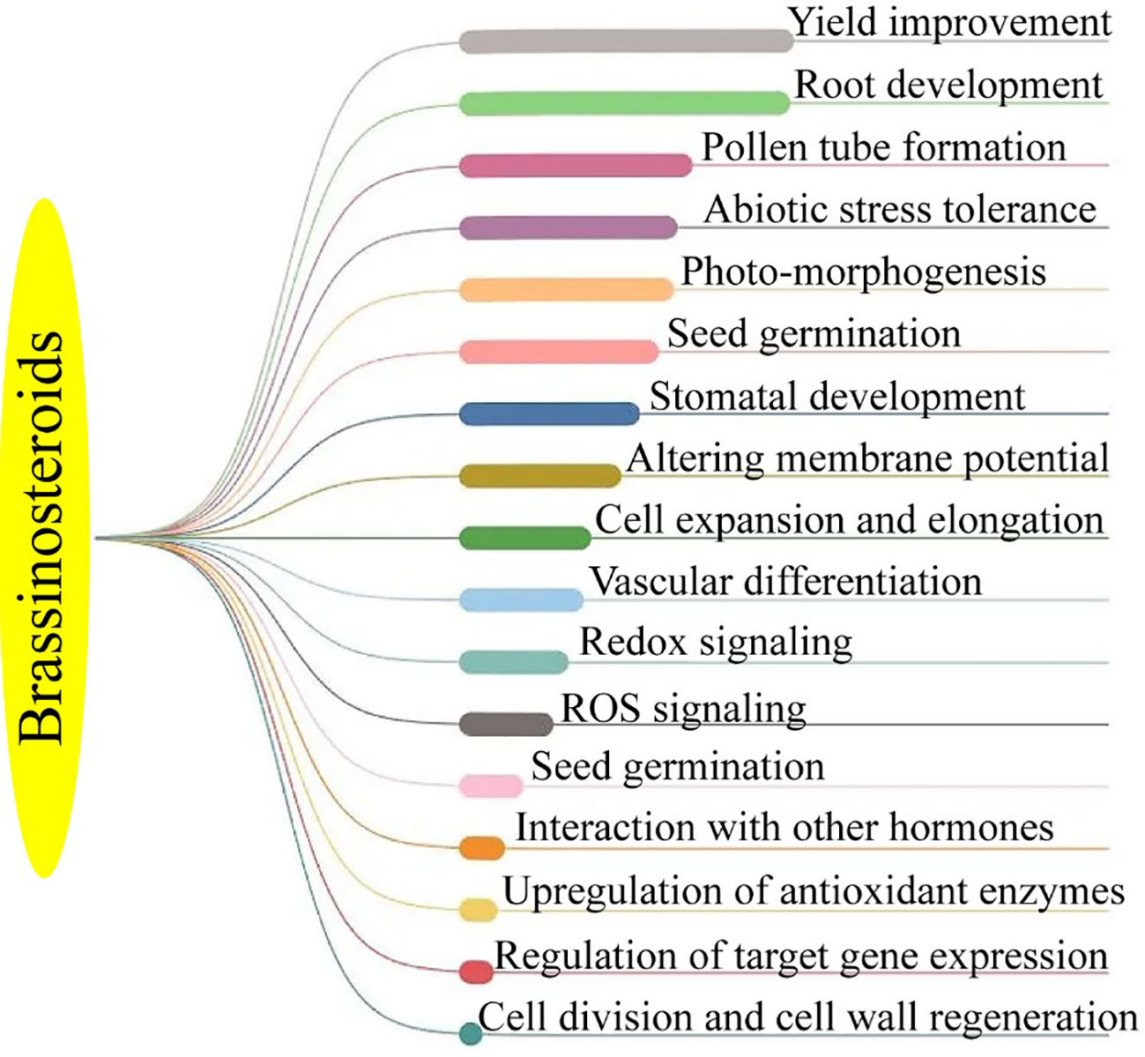
Functions of brassinosteroid in horticultural plants.

**Figure 3:**
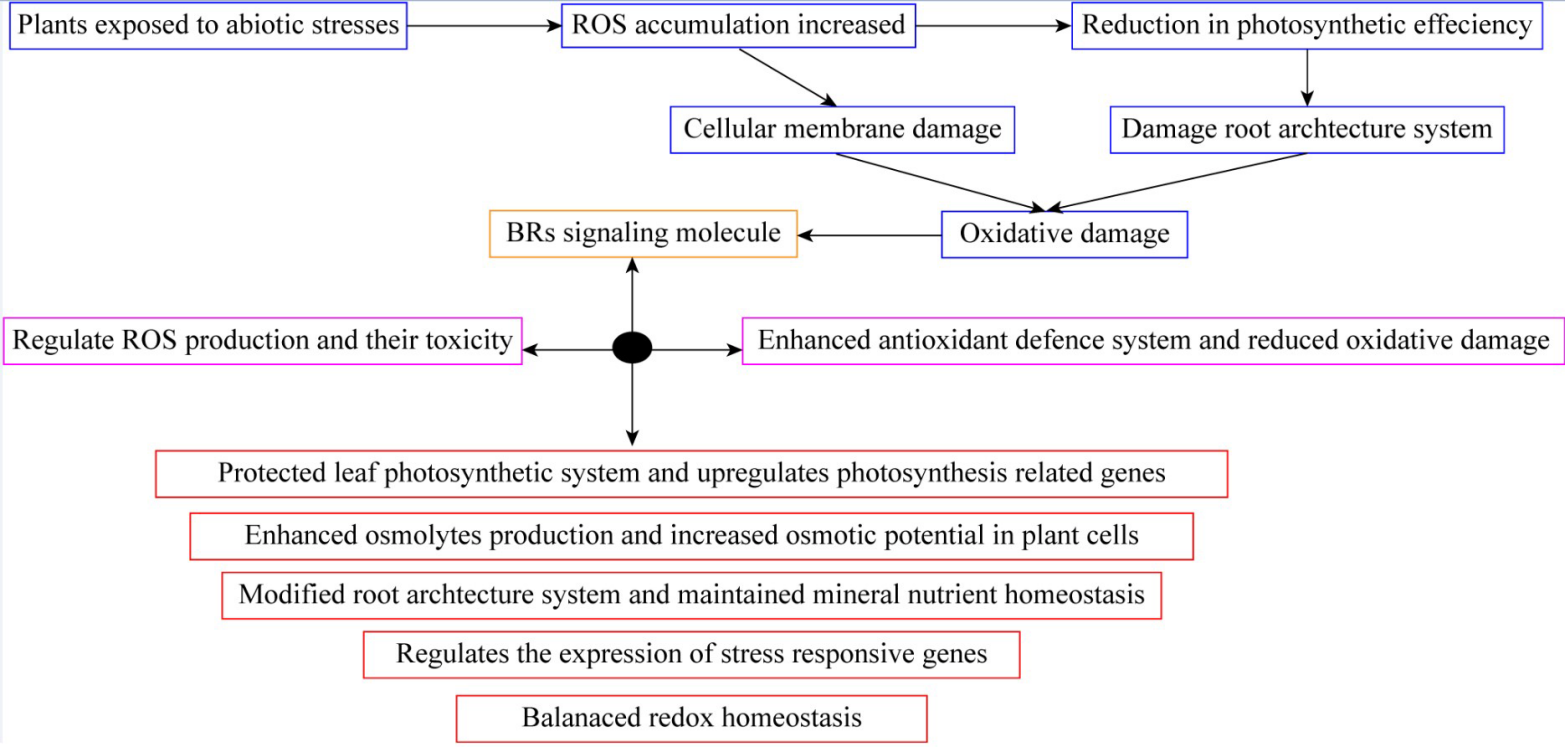
Brassinosteroid application protected leaf photosynthetic system and reduced oxidative damage in plants.

The BRs are a class of naturally occurring plant steroids that play a key role in a wide range of biological and cellular mechanisms, which include stem elongation, pollen tube progression, leaf twisting and subscale, root suppression, fruit ripening, ethylene synthesis, proton pump occurrence, xylem segmentation, chlorophyll content, and expression levels (Bajguz, 2011; Ahmad et al., 2023). Since the 1980s, research has been carried out to look into the potential economic advantages of BRs in horticultural crops. The chemical synthesis of BRs analogs then provides a way for the commercial manufacture of active BRs for greenhouse and field studies, confirming structure-activity connections (Altmann, 1999; Rhaman et al., 2020). 24-epibrassinolide (EBR) and 28-homobrassinolide (HBR) have been recognized as plant growth regulators (**Figure 2**). An extensive study on EBR and HBR studied that exogenously applied BRs can significantly boost yield and quality in several plant species (Tarkowská and Strnad, 2017). However, the outcomes can vary depending on the useful way, development stage, and external factors (**Figure 3**). Plants had potential to withstand environmental challenges such as water deficit conditions, salinity stress, and low and high temperatures due to BRs applications (Krishna, 2003). The extra benefit of using BRs in agriculture to increase agricultural output is their capacity to endow plants with resilience to abiotic stressors (Baghel et al., 2019). The role of BRs in protecting plants against environmental stresses is more vital for sustainable production (Ahmad et al., 2023). Future farming will benefit much economically from the merging of two qualities (growth stimulation and stress resistance) as transmitted by BRs for increased crop yield (Altmann, 1998; Sultan and Raza, 2015).

The variations in plant form, size, color, flavor, and aroma as well as flower or fruit color also contribute to the diversity and value of these plants (Ahmad and Anjum, 2023). The sectors of horticulture and the related green industries are professionally expanding quickly and are becoming more and more significant to society (Bajguz, 2012). It is also well-recognized that horticultural plant cultivation is characterized by rigorous and highest management costs, climate control, extensive technological utilization, and serious risk (Pereira-Netto et al., 2006). Therefore, the main objective of this review is to describe the function of BRs, a form of harmless and ecologically friendly hormone, on horticultural crops to satisfy the rise of the industrial sectors. The practical implementation of BRs in horticultural crops for improving yield, quality, and stress tolerance may have a promising future with the advancement of chemical synthesis capabilities (Tang et al., 2016; Chaudhary et al., 2023; Sardar et al., 2023).

The adoption of management strategies to increase plant yield while cultivating under abiotic stressors was advised by plant researchers. Exogenous phytohormone spray is a more attractive method to counteract the detrimental effects of abiotic stresses on long-term plant yield (Souza et al., 2017). Plant researchers are paying close attention to hormones because of their multifaceted behavior against various adverse environmental factors. For increased yields, their usage in horticultural crops growing under abiotic stressors is beneficial. Due to the multiple environmental challenges that horticulture crops face, the current study explores how phytohormones are utilized in these crops. There were also in-depth analyses of the physicochemical and molecular programming that gives plants the ability to fight against the harmful impacts of climatic extremes.

## Impact of stressors on horticultural crops

A group of polyhydroxylated steroidal plant hormones is known as BRs, involved in a wide range of physiological, biochemical, and molecular reactions in plants, including germination of seeds, cell growth and elongation, capillary distinctions, photomorphogenesis, chlorophyll content, enzyme activation, metabolism, and inhibition of cells are widely distributed all over the kingdom Plantae (Vives-Peris et al., 2017). Furthermore, it has been revealed that they can shield plants against several biotic and abiotic stresses, including pathogens, toxic substances, water, heat, and salt. Similarly, BRs increase the productivity of a majority of horticultural crops. They also improve fruit quality and yield in certain plants with significant horticultural value. Despite the fact that there are not as many of these reports, a thorough review of them has been done and is reviewed here (Khan et al., 2020). BRs control the regulation of photosynthesis, root extension, stomatal growth, leaf senescence, chlorophyll breakdown, and nutritional balance (Daszkowska-Golec, 2011; Kumar et al., 2016). It is well known that BRs play a critical role in how effectively plants can withstand stress and acclimatize to it. It’s interesting to note that BRs boost plants’ tolerance to environmental stressors. Through ROS-mediated oxidative damage control and an improved antioxidative defense strategy, exogenous BRs promoted cold stress resistance in peach (Wang X. et al., 2019). The use of BRs improved the antioxidant status of grape seedlings under high temperature conditions (Khan et al., 2020). In bitter melon under salt stress, BRs successfully enhanced growth traits, proline, and metabolite levels, and decreased oxidative injury (Souza et al., 2017). Previous research showed that BRs positively influenced strawberry pigments content, strawberry root development, cauliflower antioxidant defense system, tomato osmolytes activities, apple ROS generation, and many other horticultural crops under an abiotic stress condition (Pereira-Netto et al., 2006; Tang et al., 2016). It also enhanced leaf gas exchange parameters, lesser ROS production, and decreased heavy metal concentrations. The most prevalent, well-known, and best-characterized substance is BRs. Once horticultural crops are subjected to abiotic challenges such as drought, salinity, cold, alkalinity, heat, and metal stress, BRs effectively regulate the defensive mechanisms of the plants (Ahmad et al., 2023). The BRs can therefore lessen a range of environmental stressful threats. Additionally, exogenous BRs assisted in raising endogenous BRs concentrations in pea plants. Due to the cooling damage index, BRs treatment lowered phenolic concentration while increasing antioxidant enzyme activity (Sardar et al., 2021). Proline content and antioxidant activity were considerably increased in citrus after BRs treatment, and ROS-induced oxidative injury was reduced (Ahmad et al., 2023). Through foliar applications of BRs under waterlogging conditions in pepper plants, the metabolic activity, root aeration, antioxidative enzyme efficiency, and osmolytes content were greatly increased, while the formation of hydroxyl free radicals, MDA, and electrolyte leakage (EL) decreased (Bajguz, 2011). As a result, BRs play a favorable role in controlling how well horticultural crops respond to abiotic stress situations (Mandava et al., 2022). In numerous studies, tomatoes have undergone BRs treatment at various periods when seeds were pre-sown, root dipping, and foliar spraying (Tarkowská and Strnad, 2017). Tomato plants grown in greenhouses produced more when pre-sowing seeds with BRs for 4 hours in a 1 ppm solution. Applying 22, 23, 24-triepibrassinolide, and 28-homobrassinolide improved tomato fruit set by 43–111%, 118–129%, and 43–111%, respectively (Sridhara et al., 2021).

## Impact of BRs against abiotic stresses

**Figure 4:**
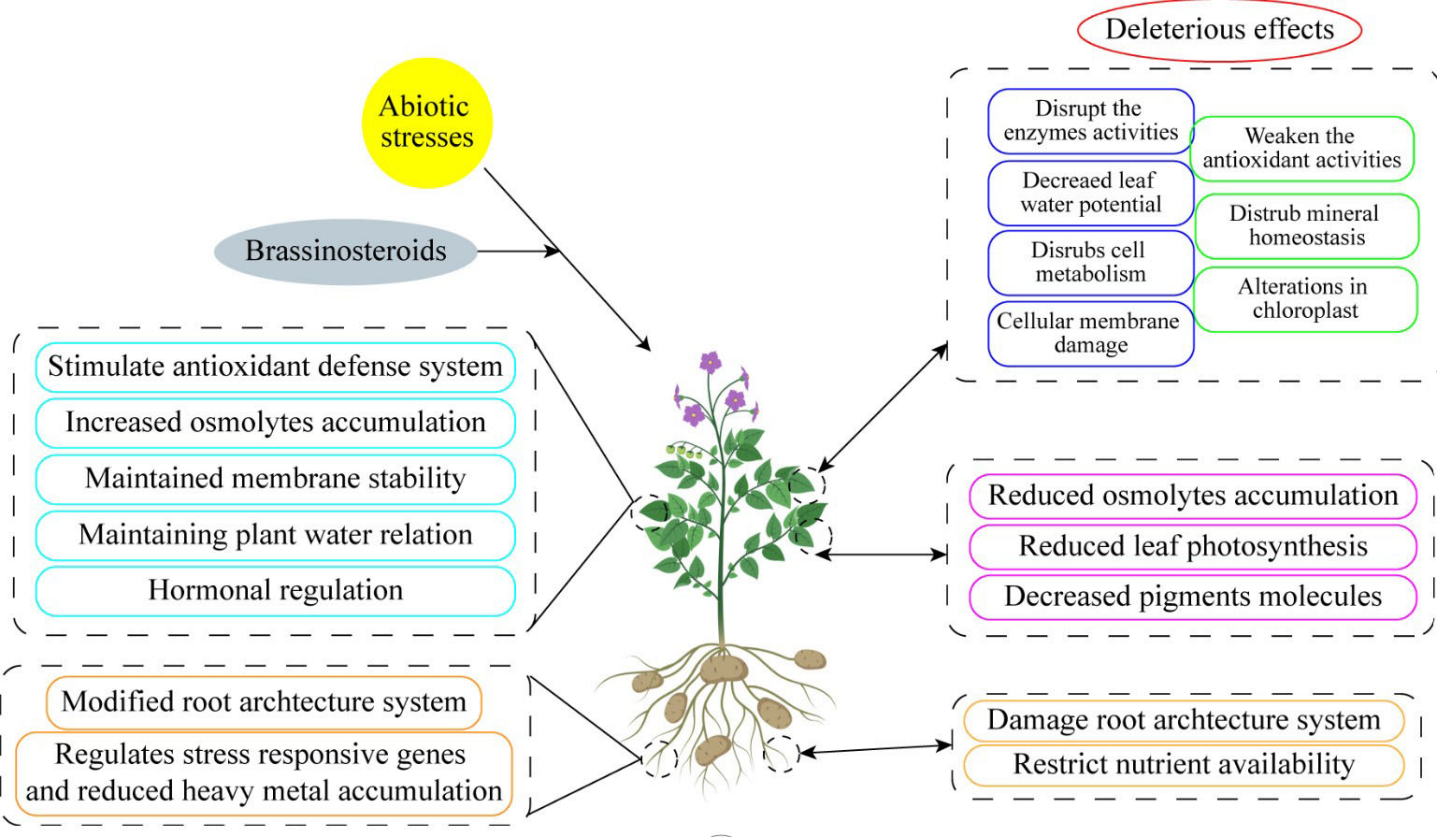
Exogenous brassinosteroid supplementation enhanced abiotic stress tolerance by increasing physiological and morphological traits.

There has been some exploration into how phytohormones concentrations and signaling status alter concerning abiotic stress for many years. (Ali et al., 2006). These variations show that phytohormones do not act as early stress signal transducers but rather as mediators of numerous upstream signals. The goal of this review is to describe the function of BRs, a type of harmless and ecologically friendly hormones, on horticultural crops in order to satisfy the evolution of the horticulture sector. Additionally, the development of biological synthesis knowledge and the actual use of BRs in horticulture crops to improve yield, quality, and stress resistance have excellent future potential (**Figure 4**). Therefore, before actually responding with BRs directly, it is imperative to look at these underlying signals (**Tables 1**–**3**).

**Table 1:** Potential functions of brassinosteroid in horticultural plants.

Functions	References
Regulated seed germination	Vardhini et al., 2006.
Modified root architecture system	Martins et al., 2017
Enhanced abiotic stress tolerance	Wei et al., 2015
Regulated stomatal development	Planas-Riverola et al., 2019
Protected photosynthetic system	Xia et al., 2009a
Upregulated antioxidant enzymes system	Wu et al., 2014
Balanced redox homeostasis	Devi et al., 2022
Cell expansion and elongation	Yang et al., 2019
Increased mineral nutrient accumulation	Singh and Savaldi-Goldstein, 2015
Reduced heavy metals accumulation	Basit et al., 2022
Enhanced Secondary metabolites accumulation	Xia et al., 2011
Fruit ripening	Wu and Yang, 2016
Flower and fruit development	Fu et al., 2008

**Table 2:** Exogenous brassinosteroid application enhanced abiotic stress tolerance in horticultural plants.

Crop name	Stress type	Reference
Peppermint	Salinity	Çoban and Baydar, 2016
Tomato	heat	Ogweno et al., 2008
pepper	Cadmium	Kaya et al., 2020
Tomato	Drought	Behnamnia et al., 2009
Tomato	Cold	Wang et al., 2022
Tomato	salinity	Zhu et al., 2016
Cucumber	Cold	Anwar et al., 2018
Pepper	Drought	Kaya et al., 2019
Cucumber	cold	Xia et al., 2009a
Lettuce	Salinity	Serna et al., 2015
Pepper	Chromium	Mumtaz et al., 2022
Radish	Cadmium	Anuradha and Rao, 2007
Orange	Cold	Ghorbani and Pakkish, 2014
Grapevine	Drought	Wang Y. et al., 2019
Tomato	Cadmium	Hayat et al., 2012
Cucumber	heat	Wei et al., 2015
Tomato	Cold	An et al., 2023

**Table 3:** Brassinosteroid enhanced physiological, morphological and metabolic processes of horticultural plants.

Crop name	Findings	References
Pepper	Reduced Cd uptake and enhanced ion homeostasis, regulates antioxidant enzymes system, leaf water potential, and decreased oxidative damage	Kaya et al., 2020
peppermint	Increased fresh and dry weight, enhanced proline level, essential oil content, antioxidant enzyme system, maintained cellular membrane integrity, secondary metabolites production	Çoban and Baydar, 2016
Cucumber	Better seedling health index, increased chlorophyll content, antioxidant enzymes activity, upregulate BRs synthesis gene expression, reduced ROS and MDA level	Anwar et al., 2018
Tomato	Regulate stress response genes, positively modulates cold stress tolerance, reduced electrolyte leakage and MDA accumulation	Wang et al., 2022
Lettuce	Decreased the adverse effect of salinity on lettuce leaf by reduced oxidative damage, and increased antioxidant enzymes activity	Serna et al., 2015
Radish	Increased seed germination rate, proline concentration, antioxidant enzymes such as SOD, GPX, CAT, APX, fresh seedling weight	Anuradha and Rao, 2007
Orange	Decreased MDA accumulation, H_2_O_2_ generation rate, increased antioxidant enzymes system, and maintained fruit quality	Ghorbani and Pakkish, 2014
Grapevine	Decreased the H_2_O_2_, and O_2_^−^ generation rate, and increased antioxidant defense system, and upregulates key defense genes	Wang Y. et al., 2019
Tomato	Regulates several antioxidant enzymes, reduced H_2_O_2_ production	Zhu et al., 2016
Pepper	Lowered chromium accumulation from root to shoot, reduced cellular membrane damage, enhanced antioxidant enzyme system and upregulates defense response genes	Mumtaz et al., 2022
Cucumber	Enhanced abiotic stress tolerance, increased seedling health index, ethylene signaling biosynthesis genes were upregulated	Wei et al., 2015

## BRs and drought

Drought is caused by a lack of water or precipitation, which significantly lowers crop yield. In locations with limited or inconsistent rainfall, the issue is worse (Ali et al., 2022). Osmotic stress, which is eventually brought on by drought, interferes with normal physiological functions by upsetting redox balance and ion allocation in the cells through absorption, extrusion, and retention (Kaya et al., 2019). The abscisic acid (ABA) buildup is intimately related to drought resistance. Exogenous BRs treatment increased ABA levels and lessen the negative impacts of drought on plants. EBR dosing in tomatoes increases drought resistance as seen by enhanced photosynthetic apparatus, leaf hydration status, and antioxidant defense in stressful conditions (Kaya et al., 2020). Exogenous BRs spray (0.02 M) in pepper seedlings can improve light use and stimulation energy absorption in the PSII antennae during dry conditions (Khamsuk et al., 2018). Exogenous BRs spray (0.1 M EBR) can improve Chorispora’s resilience to polyethylene glycol (PEG) treatment-induced dehydration (Devi et al., 2022). The transcript of genes that code for both structural and regulatory proteins is changed by BRs treatment. BRs-induced higher drought resistance in canola plants is partially attributed to EBR-induced elevated transcript levels of BnCBF5 and BnDREB (two important drought-sensitive genes) (Li J. et al., 2015). BRs interventions can lessen the long-term effects of drought on plants (Nawaz et al., 2017). Despite 60 days following a week-long water deficit, Indian mustard seedlings still showed low growth and photosynthesis (Naz et al., 2022). Nevertheless, after 30 days of sowing, treatment with 28-homobrassinolide (0.01 µM) might significantly enhance both growth and photosynthesis. While drought stress causes an excessive production of ROS, BRs intervention can significantly lower ROS, MDA, and lipid peroxidation levels when drought stress is present (Sirhindi et al., 2017). When BRs are applied exogenously, tolerance to various abiotic stimuli, such as drought, is increased. Both BR-deficient and BR-insensitive mutants exhibit increased stress tolerance (Samancıoğlu et al., 2014). Moreover, a study on tomatoes revealed that drought tolerance is improved by an increase in endogenous BRs level but not BRs signaling potency. The study also found that tomato drought tolerance was negatively impacted by BRI1 overexpression, indicating that abnormalities in the BRs network may either enhance or reduce stress tolerance, illustrating the complexity of the connections between stressors and BRs (Lv et al., 2020).

## BRs and salinity

Salinity is a significant contributor to osmotic stress, also known as physiological drought (Yin et al., 2010). Crop growth, yield, and development are all adversely affected (Zhang et al., 2016). With regards to a broad range of plants, including *Arabidopsis*, pepper, cucumber, common bean, and black locust, BRs have been established to lessen the harmful impacts of salinity (Kumari et al., 2021). The elevated antioxidant enzyme activities, reduced Na^+^ and Cl^-^, and improved K^+^ and Ca^2+^ levels in eggplants are signs of the treatment’s ability to boost their tolerance to saline conditions (Sardar et al., 2023). EBR treatment can lower NO_3_ and NH_4_ concentrations in cucumber plants that are under saline conditions (Naz et al., 2022). However, at 30 and 45 days after sowing, foliar treatment of HBL to rapeseed may successfully mitigate the negative effects of salinity stress (Zhang et al., 2022). Higher photosynthesis, nitrogen efficiencies, and total polyamines are all linked to cucumber plants improved BR-induced resistance to the saline condition (Wei et al., 2015). Exogenous EBR exposure enhanced the net rate of photosynthesis, stomatal regulation, evapotranspiration, net photosynthetic, and maximal quantum yield of PSII under the saline condition by lowering leaf Na^+^ levels and membrane permeability (Chaudhary et al., 2023; Sardar et al., 2023). BRs is also efficient in minimizing the effects of several stresses on plants. EBR (1 µM) can reduce simultaneous stress brought on by NaCl in *Brassica* species, while HBL (0.01 µM) can reduce combined stress brought on by salt and elevated heat in mung bean (Fu et al., 2008). This wide range in BRs concentrations significantly emphasized the dose-effect relationship between BRs and plant species (Cui et al., 2011). It has been demonstrated that ubiquitin-conjugating enzymes (UBC 32) have a function in BR-induced salt stress resistance. UBC32 affects the BRI1 receptor’s accumulation in cells as a functional member of the endoplasmic reticulum-associated protein degradation (ERAD) pathway, and also guides the ERAD pathway towards BR-enhanced osmotic adjustment in *Arabidopsis* (Chi et al., 2016). Additionally, BRs has been regulating DNA methylation, which is crucial for salt tolerance. A role for BRs in epigenetic alteration under salinity stress is suggested by the fact that seed priming with EBR raises total methylation and enhances salt tolerance (Wang et al., 2011).

## BRs and waterlogging

Plants faced serious injuries at the post-waterlogging stage due to a rapid flow in oxygen as reported in the peas (Jackson, 1979). Reoxygenation also referred to as post-waterlogging injuries, is the basic way of this harm. When suddenly exposed again to ambient oxygen, plants overproduce ROS as a result of reoxygenation. Photo-inhibition, or high light after water stress, harms the photosynthetic machinery (Najeeb et al., 2015). The formation of ROS in photosystem I and photosystem II, which are located in the thylakoids of the plastid, is significantly correlated with the deactivation of the photosynthesis. High light can also obliterate photosystem II directly, increasing H_2_O_2_ production. ROS also limits the production of new proteins necessary for rebuilding photo-damaged PSII (Bhusal et al., 2020). The four primary organelles for ROS generation in plants are the plastid, cellular organelles, endosomes, and endoplasmic reticulum (ER). In addition to these, enzymes for the production of ROS are also present in the apoplast, cell wall, and cell membrane. Similarly, ROS are usually created in plant metabolic activities. Singlet oxygen (1O_2_), superoxide anion (O_2_), hydrogen peroxide (H_2_O_2_), hydroxyl radical (HO), hydroperoxyl radical (HO_2_), and ozone are some of the radical and non-radical ROS found in plants (Geldhof et al., 2022). Cellular proteins, lipids, and nucleic acids can all be harmed by ROS. Although a multiplicity of plant hormones is in charge of controlling reoxygenation or dehydration damage, little is known about how these hormones interact when plants are under post-waterlogging stress (Jiao et al., 2023). But the key point is that all of these hormones work to overcome reoxygenation or dehydration stress in plants under post-waterlogging conditions, either by opening or closing stomata or by detoxifying ROS through various physiological and signaling mechanisms (Zhu et al., 2018).

## BRs and temperature

In many parts of the world, chilling or freezing damage caused by low temperatures is a serious hindrance to agricultural production, especially for thermophilic plants (Dong et al., 2021). Adjustments to membrane fluidity, changes in macromolecule interactions, a reduction in cell osmotic pressure, as well as mechanical restrictions, are all consequences of cold stress in plants. The effects of cold exposure on plant photosynthetic activities include a decrease in the pace of CO_2_ assimilation, photo-inhibition at PSI and PSII, and a decline in enzyme activity (Ma et al., 2021). The increased cold tolerance caused by BRs does not just affect whole plants but also harvested plant products like fruits. According to studies, BRs can increase the shelf life under low-temperature stress, enhancing the post-harvest integrity of horticultural crops (Zhao et al., 2019). However, compared to the amounts that are utilized to impart stress resistance in the complete plant, relatively large quantities of EBR are applied for post-harvest regulation. Fruit integrity of tomatoes is severely degraded by cold stress. However, 6 µM EBR treatment decreased the chilling-induced damage on tomato fruits (Chang and Reed, 2000; Lopez et al., 2011). Mango fruits are protected from cold-related damage by 10 mM EBR treatment by having higher amounts of a group of proteins (remorin, type II SK2 dehydrin, and temperature-induced lipocalin) (Li et al., 2012). In contrast, BRs elevated the unsaturated fatty acids in the plasma membrane phospholipids of mango fruits, lowering the phase transition temperatures and increasing fluidity under coldness (Nham et al., 2017; Shi et al., 2019). The impact of various EBR levels on the freshness of peppers at low temperatures (3°C) has been explored. Moreover, 15 µM EBR was sufficient to reduce adverse effects of chilling on the green sweet peppers (Raza et al., 2023). BRs supplementation on peppers improved antioxidant activity and photosynthetic pigments and L-ascorbic acid and expected to reduce oxidative damage and EL under cold stress (Gisbert-Mullor et al., 2021). The metabolic process that is most vulnerable to heat stress is photosynthetic machinery. High temperatures hinder photosystem II related to photochemical activities in addition to reducing net photosynthetic rate (Li T. et al., 2015). By enhancing the antioxidant enzyme functions that reduce oxidative damage under distress, EBR (0.2 µM) pretreatment in tomato can reduce losses in photosynthesis brought on by high temperatures. It is fascinating to note that BRs can regulates thermo-tolerance in plant cultivars that are both heat-tolerant and heat-sensitive (Zhang et al., 2013). In the case of both heat-tolerant and heat-sensitive ecotypes of melon, EBR pretreatment significantly increases the photosynthetic pigment levels, net CO_2_ absorption rate, stomatal closure, photo-degradation activity of PSI, and water-use efficiency under heat exposure (Raza et al., 2023). By boosting antioxidant capability, EBR treatment (0.05 - 0.2 mM) reduces heat stress in eggplant and, reduces the amount of ROS that accumulates when temperatures are high (Mohapatra et al., 2022). According to these results, BRs have specific modulatory impacts on plant photosynthesis and antioxidant properties, which significantly help to mitigate the harmful consequences of heat stress.

## BRs and heavy metals

**Figure 5:**
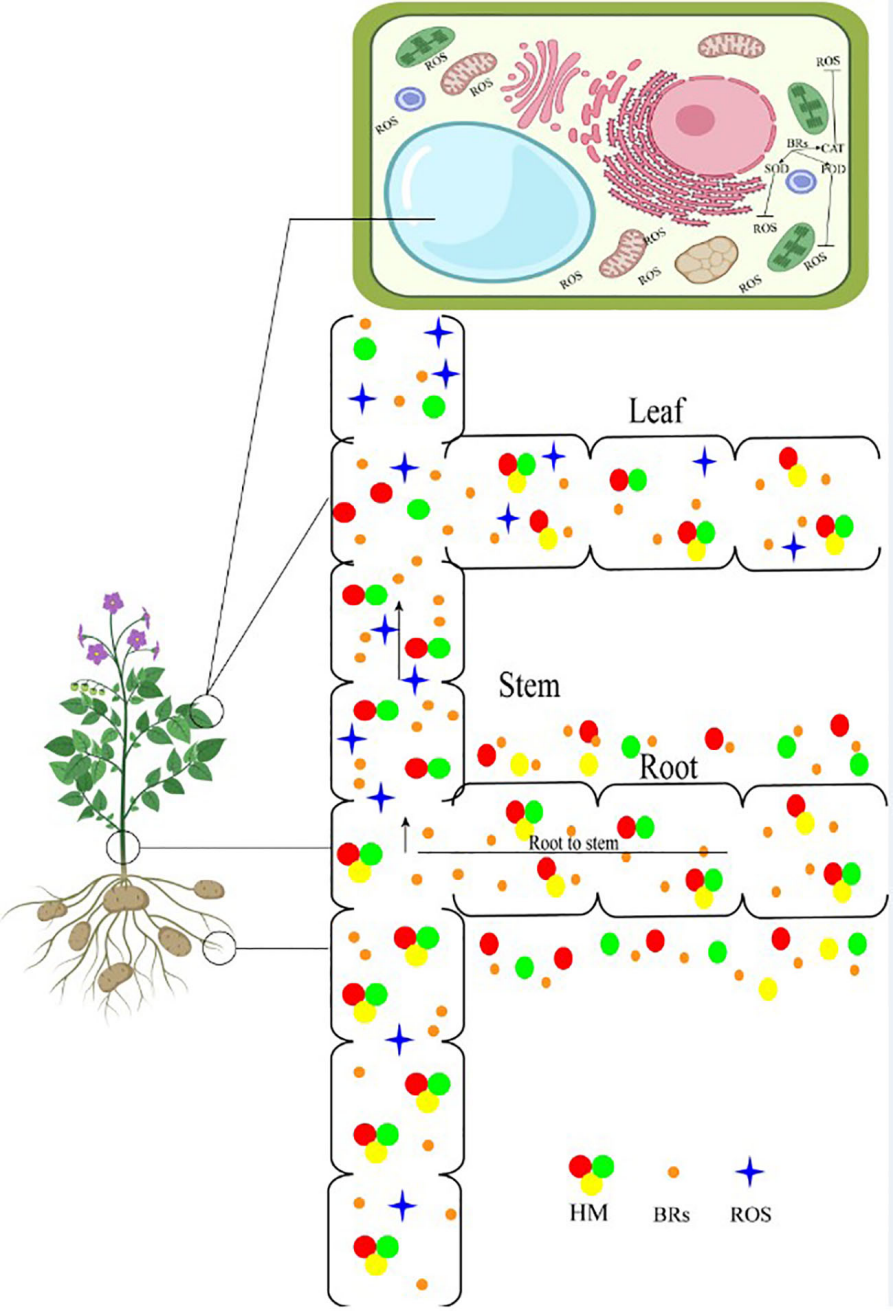
Brassinosteroid application reduced heavy metals accumulation from root to shoot. HM, heavy metals; ROS, reactive oxygen species; BRs, brassinosteroids.

Metal-induced stress is a problem for plants cultivated in contaminated soil. Stress brought on by heavy metals has certain distinctive impacts. First off, the quantity and quality of crops grown in heavy metal-contaminated soils are harmed. Second, due to possible food chain contaminants, there are severe dangers connected to consuming heavy metal-contaminated natural substances (Bücker-Neto et al., 2017). High levels of harmful metals are frequently found in crops cultivated in such metal-affected soils, and eating these contaminated foods carries additional dangers. Numerous studies using different methodologies were carried out to address these problems (Sytar et al., 2019). Heavy metal stress can be reduced through the application of plant growth regulators, bioactive substances, and modification of endogenous hormones and signaling pathways. Similarly, BRs can reduce the effects of heavy metal stress on a variety of plant species (Singh et al., 2016) (**Figure 5**). The ability of plants to absorb CO_2_ and their ability to photosynthesize are significantly impacted by heavy metals. Increasing data indicate that heavy metals like Cd reduce photosynthetic activity by restricting the Calvin cycle’s use of ATP and NADPH (Rady and Osman, 2012.). The net photosynthetic activity, stomatal functioning, maximum quantum yield of PSII, the quantum efficiency of PSII, and photochemical quenching coefficient were all considerably reduced in tomato under cadmium (Cd) stress for 40 days. BRs regulates net photosynthetic rate, restrictions of Cd accumulation in leaves, and improves CO_2_ absorption ability (Ahammed et al., 2013). As a result, Cd stress severely inhibits the formation of biomass in plants. Nevertheless, foliar EBR (0.1 M) exposure considerably boosts CO_2_ absorption capability, Fv/Fm, and total chlorophyll content during Cd stress (Vardhini, 2016). A foliar spray of Exogenous BRs also reduces Cd absorption in the roots and its transfer toward leaves. Under chromium (Cr) exposure, tobacco leaf mesophyll cells’ transmission electron micrographs revealed a deformed cellular structure and cytoplasmic membrane as well as dilated thylakoid (Jan et al., 2020). However, EBR treatment aided to maintain the organization of grana and thylakoids under Cr stress and safeguarded the chloroplast from Cr-induced injury. EBR exhibits a stress-protective function in reducing the stress caused by heavy metals. Treatment with HBL could reduce the effects of Cd on tomato seedlings’ development, photosynthetic capacity, and PSII redox reactions (Basit et al., 2022). Heavy metal exposure causes plants to produce ROS at the microscopic level, which severely affects metabolic reactions and results in oxidative damage to proteins, lipids, and biomolecules. It’s interesting to observe that plants produce more distinct BRs when exposed to heavy metals like nickel (Ni). It has been demonstrated that BRs protect plants from stress brought on by heavy metals (Fariduddin et al., 2011). Moreover, EBR treatment (0.1 M) in tomato plants can increase adaptability to Cd stress. Moreover, in soils containing less than 12 mg kg-1 Cd, foliar treatment of BRs (0.01 M EBR) can increase tomato fruit yields and quality. Within a short period of exposure, BRs have a potent protective effect against Cd stress (Yang et al., 2019). For contrast, under a 60-day Cd load, a single foliar dose of EBR (0.01 mM) applied 24 hours before the assessment can significantly increase the photosynthetic processes in tomato leaves. Leguminous crops treated with BRs have better nodule production when exposed to heavy metals. *Vigna radiata* plant development especially nodulation was disturbed under Ni stress (Posmyk and Janas, 2007). Similarly, HBL treatment reduced Cd phytotoxicity by increasing *Cicer arietinum* concentrations of both enzymatic and non-enzymatic antioxidants (Xia et al., 2009b). By increased levels of antioxidant enzymes and redox balance, the addition of EBR (5 nM) in the half-strength MS medium increases the resilience of tomato seedlings to zinc-oxide nanoparticle-induced stress (Ahmed et al., 2021). Likewise, increased endurance to heavy metals caused by exogenous BRs is considered to be due to significant improvements in photosynthetic pigments, antioxidative defense, ROS scavenging capabilities, glutathione content, phytochelatin content, and cell cycle under stress conditions (Bajguz and Hayat, 2009). So, it has been assumed that BRs had protecting role against heavy metals stress. BRs have shown great potential in reducing the detrimental effects of abiotic stress, including vanadium (V) and chromium (Cr) stress, on plants. V and Cr are heavy metals that can accumulate in plants, leading to oxidative stress and disruption of cellular processes. Studies have demonstrated that BRs can alleviate V and Cr stress by enhancing antioxidant defense systems, regulating ROS scavenging, and promoting the synthesis of stress-related proteins (Fariduddin et al., 2011). BRs also play a crucial role in maintaining cellular ion homeostasis and restricting metal accumulation in plants. The application of BRs offers a promising strategy to mitigate the toxic effects of V and Cr stress on plant growth and development, contributing to improved crop productivity and environmental sustainability (Ahammed et al., 2013).

## Future scenarios

Enhancing plants’ capacity to survive abiotic stress is crucial, and there is still a lot to be done to protect subsequent generations from the impending problem. It is possible in many ways.

◼ For pollution lessening, it is necessary to stop global warming. In this regard, conferences should be held on a national and international scales, the public should be made aware of the issue, and industries should be built according to a plan.
◼ The mechanism of stress tolerance should be explored to identify the crucial components contributing to drought response, such as the function of phytohormones and the genes expression.
◼ Transgenic developments should be used for the development of climate-resilient cultivars.
◼ It is important to test stress-tolerant cultivars in the field rather than just in the lab and greenhouses. Together, these laws and innovations will assist plants in better withstanding climate change and combating abiotic stress.

## Conclusion

BRs are a class of steroidal plant hormones that are produced naturally by all members of the plant family. There are several BRs analogues, but epibrassinolide, homobrassinolide, and brassinolide are the most stable ones. However, there are also a few synthetic and commercial substitutes of BRs in the market. BRs are allegedly non-toxic and environmentally safe. They are widely used to increase the yield of several crops. However, there has been very little research conducted on this broad topic, which has mostly gone unexplored. Horticultural crops can benefit from the huge potential of BRs by increasing yield. Since BRs are also well known for their function in protecting plants against biotic stress scenarios like the attack of various pathogens, which includes a variety of environmental stresses. As a result, they can quickly and effectively replace various pesticides and fungicides that pose a risk to human health and harm the environment.

## Author contributions

ZZ and ZC: Conceptualization, Literature survey, Writing major original draft, Review structure. HS: Writing- review and editing, Figure designing. SC: Reviewing and editing, References collection. All authors contributed to the article and approved the submitted version.
